# A Novel Reconstruction Algorithm with High Performance for Compressed Ultrafast Imaging

**DOI:** 10.3390/s22197372

**Published:** 2022-09-28

**Authors:** Qian Shen, Jinshou Tian, Chengquan Pei

**Affiliations:** 1 Key Laboratory of Ultra-Fast Photoelectric Diagnostics Technology, Xi’an Institute of Optics and Precision Mechanics, Xi’an 710049, China; 2 School of Optoelectronics, University of Chinese Academy of Sciences, Beijing 100049, China; 3 School of Artificial Intelligence, Xidian University, Xi’an 710071, China

**Keywords:** compressed ultrafast photography, computational imaging, intelligent reconstruction algorithm

## Abstract

Compressed ultrafast photography (CUP) is a type of two-dimensional (2D) imaging technique to observe ultrafast processes. Intelligence reconstruction methods that influence the imaging quality are an essential part of a CUP system. However, existing reconstruction algorithms mostly rely on image priors and complex parameter spaces. Therefore, it usually takes a lot of time to obtain acceptable reconstruction results, which limits the practical application of the CUP. In this paper, we proposed a novel reconstruction algorithm named PnP-FFDNet, which can provide a high quality and high efficiency compared to previous methods. First, we built a forward model of the CUP and three sub-optimization problems were obtained using the alternating direction multiplier method (ADMM), and the closed-form solution of the first sub-optimization problem was derived. Secondly, inspired by the PnP-ADMM framework, we used an advanced denoising algorithm based on a neural network named FFDNet to solve the second sub-optimization problem. On the real CUP data, PSNR and SSIM are improved by an average of 3 dB and 0.06, respectively, compared with traditional algorithms. Both on the benchmark dataset and on the real CUP data, the proposed method reduces the running time by an average of about 96% over state-of-the-art algorithms, and show comparable visual results, but in a much shorter running time.

## 1. Introduction

Compressed ultrafast photography (CUP) is a new ultrafast computational imaging method, and it can achieve an imaging speed of 10^11^ frames per second within a single shot. This technology introduces the framework of compressed sensing [1] into the imaging process of streak cameras, extending the imaging capability of streak cameras from one-dimensional (1D) to two-dimensional (2D). This emerging 2D ultrafast imaging technique is of great significance in revealing the fundamental mechanisms of physics, chemistry, and biomolecules [2]. It has a wide range of applications in the fields of fluorescence lifetime detection [3], real-time visualization of laser dynamics [4,5], and wide-field time-of-flight volume imaging [6], et al.

CUP reconstruction refers to establishing an inverse problem model based on the compressed sensing framework, and using an iterative algorithm to reconstruct 3D video data from the compressed 2D image captured by CUP. The reconstruction algorithm used in previous works in CUP is the two-step iterative threshold method (TwIST) [7]. Using total variation (TV) regularization in the TwIST reconstruction algorithm can easily induce artifacts [8], which limits the spatial-temporal resolution of CUP imaging and reconstruction efficiency. Ref. [9] introduced spatial and intensity constraints in the original TwIST algorithm by using an additional charge-coupled device (CCD) camera in the experimental system to reduce low-intensity artifacts. Ref. [10] proposed applying the plug-and-play alternating direction method of multipliers (PnP-ADMM) [11] to the CUP reconstruction problem, and the block-matching 3D filtering denoising (BM3D) [12] algorithm is applied to the solution of the sub-problem of this algorithm, which improves the quality of the reconstruction and effectively suppresses the resolution anisotropy and artifacts. However, the variable separation strategy adopted does not take full advantage of the fact that the sensing matrix is a block-diagonal matrix, and the convergence of the PnP-ADMM algorithm applied to the CUP reconstruction problem is not explained. When applying the BM3D algorithm to video data, it is necessary to perform BM3D denoising for each frame, which is very time-consuming, while denoising algorithms based on neural networks have better denoising performance in computing speed.

The alternating direction multiplier method (ADMM) is a widely used algorithm for constrained optimization problems in image restoration. Based on ADMM, the PnP-ADMM algorithm implements a modular structure. The biggest advantage of PnP-ADMM is that it allows the state-of-the-art denoising algorithms to be applied to the solution process of sub-optimization problems without specifying specific priors, which greatly improves the flexibility of the algorithm. Therefore, an excellent denoising algorithm can be found and inserted into the algorithm framework of PnP-ADMM to improve the reconstruction quality of the algorithm. Existing image denoising algorithms can be divided into two categories: model-based methods and discriminative-learning-based methods. Similar to total variation denoising (TVD) [13], BM3D, and weighted nuclear norm minimization (WNNM) [14] for image denoising, these algorithms are flexible in dealing with denoising problems with different noise levels, but they have some drawbacks. For example, algorithms are generally time-consuming and have many parameters that need to be manually tuned. Furthermore, these algorithms usually rely on manually determined priors, such as sparsity [15,16] and non-local self-similarity [17,18,19], which have limitations for describing complex image structures. Discriminant learning has been widely studied in image denoising due to its advantages of fast inference speed and good performance. Some examples include learning deep CNN denoiser prior for image restoration (IRCnn) [20], deep CNN for image denoising (DnCnn) [21], and fast and flexible solution for CNN-based image denoising (FFDNet) [22]. Their non-linear mapping layer is a collection of “Convolution + Batch Normalization + Rectified Linear Units” layers with filters of spatial size 3 × 3. Among them, FFDNet has several desirable properties that make it very suitable as a denoiser to be applied to the framework of PnP-FFDNet. First, it introduces a noise map as an input channel, so that a single model can handle a wide range of noise levels, so that it exhibits apparent results on both synthetic noisy images corrupted by additive white Gaussian noise (AWGN) and real-world noisy images [22,23]. Second, FFDNet reduces the size of the input by down-sampling, which makes it faster for forward inference. Therefore, compared with the model-based algorithm BM3D, with excellent de-noising performance even on CPU, FFDNet is faster without sacrificing the denoising performance. These properties make FFDNet very suitable as a denoiser in the framework of PnP-ADMM. In this paper, we proposed a novel reconstruction algorithm based on PnP and FFDNet to reconstruct the CUP system. FFDNet can learn the noise model in CUP system well. The performance on both simulated datasets and real data shows that our method performs well on both reconstructed visual effects and metric evaluation, and the reconstruction time is greatly reduced.

## 2. Forward Model of CUP

### 2.1. Principle of Streak Camera

**Figure 1:**
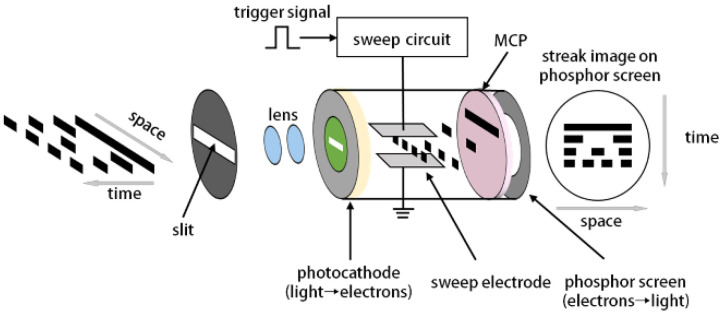
Schematic diagram of the imaging process of the streak camera in one-dimensional view.

Streak camera is an ultrafast imaging device that can capture dynamic events that occur on picosecond or even femtosecond timescales. As shown in Figure 1, a long slit with width of several microns is usually inserted in front of streak camera. The optical signal is converted into an electrical signal through the streak image tube and space-time mapping is performed by an ultrafast scanning unit. Microchannel plate (MCP) realizes the multiplication of photoelectrons, and the phosphor screen converts the photoelectrons into optical signals.

### 2.2. Design of Compressed Ultrafast Photography

**Figure 2:**
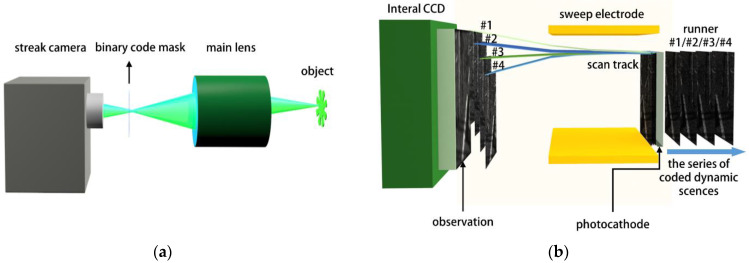
Schematic diagram of the CUP system. (**a**) Schematic diagram of the optical path structure of the CUP. (**b**) Schematic diagram of the time series of the encoded dynamic scene being shifted after entering the streak camera.

Figure 2a shows a schematic diagram of the CUP imaging system. The experimental system is mainly composed of streak camera (integrated CCD), a random binary mask, and a main camera lens. The random binary mask performs intensity modulation on the image of the detection target passing through the main lens. The light intensity modulation factor of the transparent area of the mask is 1, and the light intensity modulation factor of the opaque area of the mask is 0. The difference between CUP and the traditional streak camera is that the CUP requires the slit on the streak camera to be fully opened. This is the key to CUP’s ability to perform 2D ultrafast imaging. Figure 2b shows how the streak camera works in the CUP. Since there is no limitation of the slit, the one to four frames of the encoded images entered into the streak camera and were scanned by an ultrafast electric field. Then, the second frame was sheared by a pixel compared with the first frame in the scanning axis, and this was also performed on the third, fourth frames, etc. Finally, all the images were accumulated into a compressed image and recorded by a CCD camera. Next, the intelligence methods were performed to reconstruct the dynamic ultrafast video from a compressed image.

**Figure 3:**
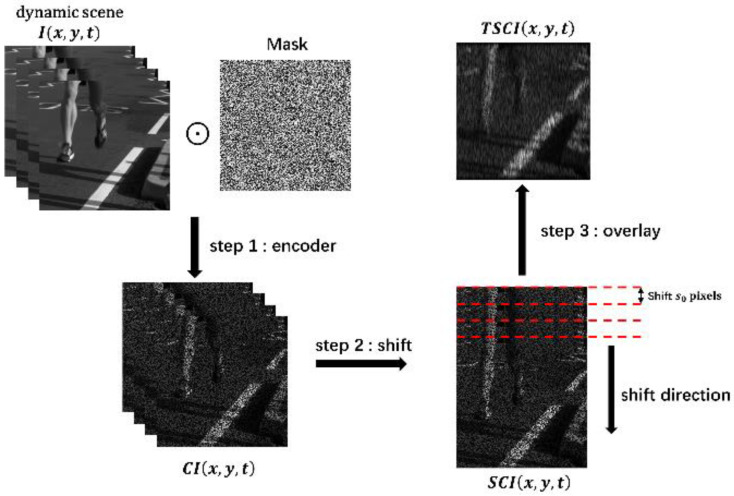
Data compression flowchart for CUP.

Figure 3 describes the basic workflow of the CUP system. Four frames are selected from the benchmark dataset runner to simulate the dynamic scene $I\left(x,y,t\right)$, with different frames simulating the moment when the dynamic scene occurs. Each frame is of size 256 × 256, and then encoded by random matrix, which is a $\left\{0,1\right\}$ randomly distributed binary code. The symbol ⊙ denotes the Hadamard (element-wise) product. The data compression of CUP follows these three steps:

Step 1: Encoding. Each frame of the dynamic scene $I\left(x,y,t\right)$ is ⊙ with the same mask, and the encoding operation is denoted as C. The encoded dynamic scene is $CI\left(x,y,t\right)$;

Step 2: Shift. A deflection electrode inside the streak camera provides a deflection voltage in the vertical direction, so the frames that arrived at different times were shifted in the vertical direction at different positions. The direction of the offset of each frame is marked in Figure 3. For the convenience of mathematical simplification later, it is assumed that each frame is offset by $s_{0}$ pixels, the shift operation is recorded as S, and the dynamic coding scene after translation is $SCI\left(x,y,t\right)$;

Step 3: Overlay. The receiver of the streak camera is an internal CCD. During the exposure time of the CCD, photons arriving at the CCD at different times are accumulated. The $SCI\left(x,y,t\right)$ in step 2 is superimposed along the time axis, denoted as T, and the two-dimensional observation result $Y\left(m,n\right)=TSCI\left(x,y,t\right)$ is finally obtained.

The goal of CUP reconstruction is to reconstruct the original three-dimensional dynamic scene $I\left(x,y,t\right)$ from the obtained two-dimensional observation $Y\left(m,n\right)$. Without loss of generality, the dynamic scene can be viewed as video data with N frames, and will mathematically describe the CUP imaging process and build a classical inverse problem model.

**Figure 4:**
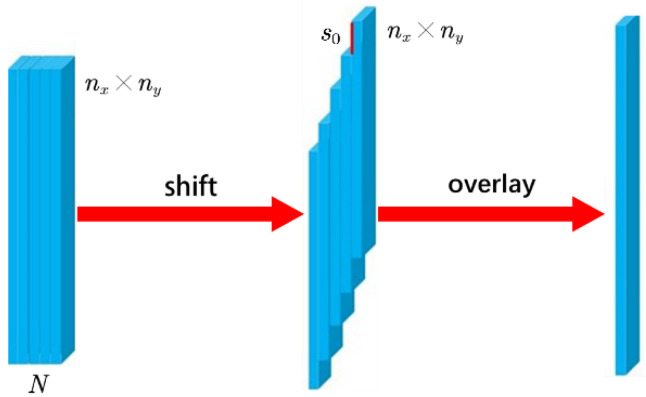
For a data cube of size $n_{x}\times n_{y}\times N$. Its height is $n_{x}$, width is $n_{y}$, and the number of frames is N. Then, the compressed image size is $[\left(N-1\right)s_{0}+n_{x}]\times n_{y}$.

**Figure 5:**
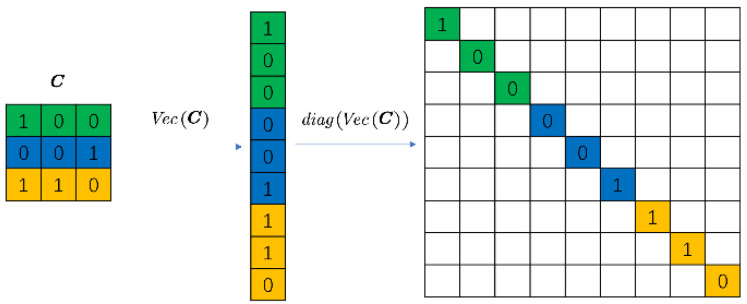
Example when mask is a 3 × 3 matrix. Different colours represent different rows of C.

The video data $X\left(X\in {\mathbb{R}}^{n_{x}\times n_{y}\times N}\right)$ that are the matrix representation of the dynamic scene $I\left(x,y,t\right)$ with N frames are compressed into one frame of two-dimensional observation data $Y\left(Y\in {\mathbb{R}}^{L\times n_{y}}\right)$, which are the matrix representation of the observation $Y\left(m,n\right)$ by the CUP system. Figure 4 shows the relationship between L and $n_{x}$. Therefore, $L=\left(N-1\right)s_{0}+n_{x}$. The coding matrix $C\left(C\in {\mathbb{R}}^{n_{x}\times n_{y}}\right)$ used by the CUP system is random binary code. The data compression observation model of CUP [10] can be expressed as:

(1) Y=TSCX+Z

where $Z\left(Z\in {\mathbb{R}}^{L\times n_{y}}\right)$ represents the noise in the observation process, $X\left(X\in {\mathbb{R}}^{n_{x}\times n_{y}\times N}\right)$ can be expressed as ${ [X_{1},X_{2},\cdots ,X_{N}]}^{T}$, C represents the encoding process, S represents the shift process, and T represents the accumulating process. T can be represented by the summation notation:

(2) $$Y=\sum_{i=1}^{N}S_{i}\left(C⊙X_{i}\right)+Z$$

where ⊙ denotes the Hadamard (element-wise) product. To convert the Hadamard product to matrix multiplication, $Y\left(Y\in {\mathbb{R}}^{L\times n_{y}}\right)$, $X\left(X\in {\mathbb{R}}^{n_{x}\times n_{y}\times N}\right)$, and $Z\left(Z\in {\mathbb{R}}^{L\times n_{y}}\right)$ are transformed into 1D column vectors:

(3) $$\begin{array}{ll} y & =\sum_{i=1}^{N}S_{i}^{\prime }(C_{diag}x_{i})+z\\ & =\sum_{i=1}^{N}(S_{i}^{\prime }C_{diag})x_{i}+z\\ & =[S_{1}^{\prime }C_{diag},S_{2}^{\prime }C_{diag},\ldots ,S_{N}^{\prime }C_{diag}]{\left[x_{1}^{T},x_{2}^{T},\ldots ,x_{N}^{T}\right]}^{T}+z \end{array}$$

where $x_{i}=Vec\left(X_{i}\right)\in {\mathbb{R}}^{n_{x}n_{y}\times 1}$, $y=Vec\left(Y\right)\in {\mathbb{R}}^{Ln_{y}\times 1}$ and $z=Vec\left(Z\right)\in {\mathbb{R}}^{Ln_{y}\times 1}$, $C_{diag}=diag\left(Vec\left(C\right)\right)\in {\mathbb{R}}^{n_{x}n_{y}\times n_{x}n_{y}}$. Figure 5 shows a simple example when the mask is 3 × 3.

**Figure 6:**
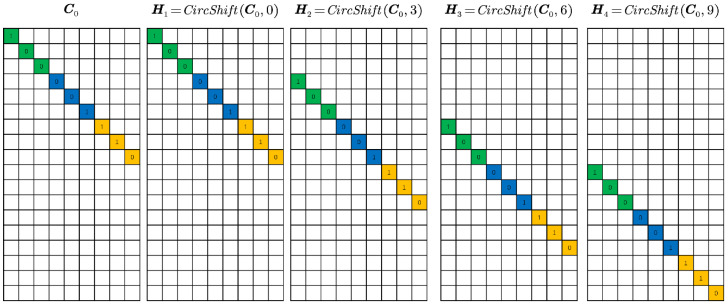
Example of the matrix H. Different colours represent different rows of C.

Equation (3) can be mathematically described as a classic inverse problem model as follows:

(4) y=Hx+z

where $x={ [x_{1}^{T},x_{2}^{T},\cdots ,x_{N}^{T}]}^{T}$, and the sensing matrix $H\in {\mathbb{R}}^{Ln_{y}\times n_{x}n_{y}N}$ is a block-diagonal matrix, H can be expressed as:

(5) $$H=[H_{1},\ldots ,H_{N}]=[S_{1}^{\prime }C_{diag},S_{2}^{\prime }C_{diag},\ldots ,S_{N}^{\prime }C_{diag}]$$

where $S_{i}^{\prime }=CircShift\left(I_{0},\left(i-1\right)s_{0}n_{y}\right)\in {\mathbb{R}}^{Ln_{y}\times n_{x}n_{y}},i=1,2,3,\ldots N$, $s_{0}$ indicates the number of pixels per shift. $CircShift\left(A,l\right)$ represents the circular translation of l pixels along the vertical direction of the matrix A. $I_{0}\in {\mathbb{R}}^{Ln_{y}\times n_{x}n_{y}}$ can be expressed as:

(6) $$I_{0}= [\begin{array}{c} I_{n_{x}n_{y}\times n_{x}n_{y}}\\ O_{\left(N-1\right)s_{0}n_{y}\times n_{x}n_{y}} \end{array}]$$

where $I\in {\mathbb{R}}^{n_{x}n_{y}\times n_{x}n_{y}}$ represents the identity matrix. A simple case of $H_{i}$ in Equation (5) when the number of frames is four is shown in Figure 6, and it can be written as: $H_{i}=CircShift\left(C_{0},\left(i-1\right)s_{0}n_{y}\right)\in {\mathbb{R}}^{Ln_{y}\times n_{x}n_{y}},i=1,2,3,\ldots N$ and $C_{0}\in {\mathbb{R}}^{Ln_{y}\times n_{x}n_{y}}$ can be expressed as:

(7) $$C_{0}= [\begin{array}{c} {\left(C_{diag}\right)}_{n_{x}n_{y}\times n_{x}n_{y}}\\ O_{\left(N-1\right)s_{0}n_{y}\times n_{x}n_{y}} \end{array}]$$

An important property of the sensing matrix is that $HH^{T}$ is a diagonal matrix, which is used in Section 3.1 to obtain the closed-form solution of the suboptimization problem. $HH^{T}$ can be written as $HH^{T}=\sum_{i=1}^{N}H_{i}H_{i}^{T}$ and consider that when i=1, the result of $H_{1}H_{1}^{T}$ is:

(8) $$H_{1}H_{1}^{T}= [\begin{array}{c} C_{diag}\\ O \end{array}] [\begin{array}{cc} C_{diag} & O \end{array}]= [\begin{array}{cc} C_{diag} & O\\ O & O \end{array}]$$

where $C_{diag}=diag{\left(Vec\left(C\right)\right)}_{n_{x}n_{y}\times n_{x}n_{y}}$ is a diagonal matrix and O represent the zero matrix. In the case of i=1, the result of $H_{1}H_{1}^{T}$ is a diagonal matrix. When generalized to i=2,⋯,N, the result of $H_{i}H_{i}^{T}$ is still a diagonal matrix, so their sum is also naturally a diagonal matrix and, thus, $HH^{T}$ is a diagonal matrix.

## 3. Novel Reconstruction Method for CUP

### 3.1. Algorithm Framework of PnP-ADMM for CUP

According to the forward model of CUP data compression already established in Section 2.2, the inverse problem model of Equation (4) can be described as an unconstrained optimization problem:

(9) $$\hat{x}=arg\underset{x}{\min}f\left(x\right)+\lambda g\left(x\right)$$

In the formula, $f\left(x\right)=\|y-Hx{\|}_{2}^{2}$ represents the CUP forward imaging model, and $g\left(x\right)$ represents a certain image prior.

The ADMM transforms the unconstrained optimization problem (9) into a constrained optimization problem by introducing an auxiliary variables v:


(10) $$\left(\hat{x},\hat{v}\right)=\arg\underset{x,v}{\min}f\left(x\right)+\lambda g\left(v\right),\mathrm{subject}\mathrm{to}x=v$$

The minimum optimization problem (10) can be solved by iteratively solving the following three sub-optimization problems [24]:

(11) $$x^{\left(k+1\right)}=arg\underset{x}{\min}f\left(x\right)+\frac{\rho }{2}\|x-\left(v^{\left(k\right)}-\frac{1}{\rho }u^{\left(k\right)}\right){\|}_{2}^{2}$$

(12) $$v^{\left(k+1\right)}=arg\underset{v}{\min}\lambda g\left(v\right)+\frac{\rho }{2}\|v-\left(x^{\left(k\right)}+\frac{1}{\rho }u^{\left(k\right)}\right){\|}_{2}^{2}$$

(13) $$u^{\left(k+1\right)}=u^{\left(k\right)}+\rho \left(x^{\left(k+1\right)}-v^{\left(k+1\right)}\right)$$

where k represents the number of iterations.

In the CUP reconstruction problem, $f\left(x\right)=\frac{1}{2}\|y-Hx{\|}_{2}^{2}$ represents the forward model of CUP imaging. For the convenience of representation, the sub-problem (11) is rewritten in the form without the iteration variable k:

(14) $$x=arg\underset{x}{\min}\frac{1}{2}\|y-Hx{\|}_{2}^{2}+\frac{\rho }{2}\|x-\left(v-\frac{1}{\rho }u\right){\|}_{2}^{2}$$

For the determined v,u,H,y, sub-problem (14) has a closed-form solution:

(15) $$x={\left(H^{⊤}H+\rho I\right)}^{-1} [H^{⊤}y+\rho \left(v-\frac{1}{\rho }u\right)]$$

For a large matrix H, inverting $\left(H^{T}H+\rho I\right)$ will consume a lot of computer memory resources and time. For the original data X with size of 256 × 256 × 8, the size of H is 67,328 × 524,288, and the size of $H^{T}H$ is 524,288 × 524,288. At present, it is difficult for computers to deal with such a large-scale matrix inversion problem. Inspired by [25], when $HH^{T}$ is a diagonal matrix, Equation (14) has a closed-form solution. It was discussed in Section 2.2 that the matrix H is a block-diagonal matrix, and it is verified that $HH^{T}$ is a diagonal matrix. This feature of H can simplify the computation of the inversion of $\left(H^{T}H+\rho I\right)$:

(16) $${\left(H^{⊤}H+\rho I\right)}^{-1}=\rho^{-1}I-\rho^{-1}H^{⊤}{\left(I+\rho^{-1}HH^{⊤}\right)}^{-1}H\rho^{-1}$$

Bringing Equation (16) into Equation (15), we can obtain [25]:

(17) $$\begin{array}{l} x=\rho^{-1} [H^{⊤}y+\rho \left(v-\frac{1}{\rho }u\right)]-\rho^{-2}H^{⊤}{\left(I+\rho^{-1}HH^{⊤}\right)}^{-1}HH^{⊤}y\\ -\rho^{-1}H^{⊤}{\left(I+\rho^{-1}HH^{⊤}\right)}^{-1}H\left(v-\frac{1}{\rho }u\right) \end{array}$$

where $HH^{T}$ is a diagonal matrix:

(18) $$HH^{⊤}=diag\left( [\psi_{1},\ldots ,\psi_{Ln_{y}}]\right)$$

then we can obtain:

(19) $${\left(I+\rho^{-1}HH^{⊤}\right)}^{-1}=diag\left( [\frac{\rho }{\rho +\psi_{1}},\ldots ,\frac{\rho }{\rho +\psi_{Ln_{y}}}]\right)$$

(20) $${\left(I+\rho^{-1}HH^{⊤}\right)}^{-1}HH^{⊤}=diag\left( [\frac{\rho \psi_{1}}{\rho +\psi_{1}},\ldots ,\frac{\rho \psi_{Ln_{y}}}{\rho +\psi_{Ln_{y}}}]\right)$$

If $y={ [y_{1},\ldots ,y_{Ln_{y}}]}^{⊤}$, ${ [H\left(v-\frac{1}{\rho }u\right)]}_{i}$ denote the i th element of vector $[H\left(v-\frac{1}{\rho }u\right)]$, Equation (17) becomes [25]:

(21) $$\begin{array}{c} x=\rho^{-1}H^{T}y+\left(v,-,\frac{1}{\rho },u\right)\\ -\rho^{-1}H^{T}{ [\frac{y_{1}\psi_{1}+\rho { [H,\left(v-\frac{1}{\rho }u\right)]}_{1}}{\rho +\psi_{1}},,,\ldots ,,,\frac{y_{Ln_{y}}\psi_{Ln_{y}}+\rho { [H,\left(v-\frac{1}{\rho }u\right)]}_{Ln_{y}}}{\rho +\psi_{Ln_{y}}}]}^{T}\\ \left(v-\frac{1}{\rho }u\right)+H^{T}{ [\frac{y_{1}-{ [H,\left(v-\frac{1}{\rho }u\right)]}_{1}}{\rho +\psi_{1}},,,\ldots ,,,\frac{y_{Ln_{y}}-{ [H,\left(v-\frac{1}{\rho }u\right)]}_{Ln_{y}}}{\rho +\psi_{Ln_{y}}}]}^{T} \end{array}$$

Using matrix division of corresponding elements, Equation (21) can be simplified to:

(22) $$x=\left(v-\frac{1}{\rho }u\right)+H^{⊤} [y-H\left(v-\frac{1}{\rho }u\right)]./\left(s+\rho I\right)$$

where $s={ [\psi_{1},\ldots ,\psi_{Ln_{y}}]}^{⊤}$, in Equation (22), the matrix division of the corresponding element has priority.

By utilizing the property that $HH^{⊤}$ is a diagonal matrix, in each iteration process, the solution of sub-optimization problem (11) can be completed with only one calculation, so the computer memory load is reduced and the solution efficiency of the algorithm is improved.

One of the most important features of ADMM iteration is its modular structure: problem (11) can be seen as a reversal step, since it includes the forward imaging model $f\left(x\right)$, and problem (12) can be seen as a denoising step, because it includes the image a priori $g\left(v\right)$. If $\sigma =\sqrt{\frac{\lambda }{\rho }}$, problem (12) can be rewritten as [24]:

(23) $$v^{\left(k+1\right)}=arg\underset{v}{\min}\lambda g\left(v\right)+\frac{1}{2\sigma^{2}}\|v-\left(x^{\left(k\right)}+\frac{1}{\rho }u^{\left(k\right)}\right){\|}_{2}^{2}$$

Considering $\left(x^{\left(k\right)}+\frac{1}{\rho }u^{\left(k\right)}\right)$ as a noisy image, problem (23) minimizes the two-norm distance between the noise-free image v and the noisy image $\left(x^{\left(k\right)}+\frac{1}{\rho }u^{\left(k\right)}\right)$, based on the image prior $g\left(v\right)$ as a regular term. If $g\left(v\right)=\|v{\|}_{TV}$, where $\|\cdot {\|}_{TV}$ represents the total variation norm, it can be calculated by Equation (24) [24]:


(24) $$\|v{\|}_{TV}=\sum_{i}\sqrt{{\left(\Delta_{i}^{h},v\right)}^{2}+{\left(\Delta_{i}^{v},v\right)}^{2}}$$

Then problem (23) becomes the standard total variation norm denoising problem, namely the TV denoising problem. Formally based on this intuition, [11] proposed the PnP-ADMM method, without specifying the image prior $g\left(v\right)$, and just replaces step (12) with a state-of-the-art image denoising algorithm [24]:

(25) $$v^{\left(k+1\right)}=D_{\sigma }\left(x^{\left(k\right)}+\frac{1}{\rho }u^{\left(k\right)}\right)$$

where $D_{\sigma }\left(\cdot \right)$ represents some type of noise denoising algorithm. Although it is unclear to which image prior the denoising algorithm $D_{\sigma }\left(\cdot \right)$ corresponds, the performance of the PnP-ADMM method on the image reconstruction problem surpasses other popular image reconstruction algorithms [26,27,28,29].

### 3.2. The Architecture of FFDNet

**Figure 7:**
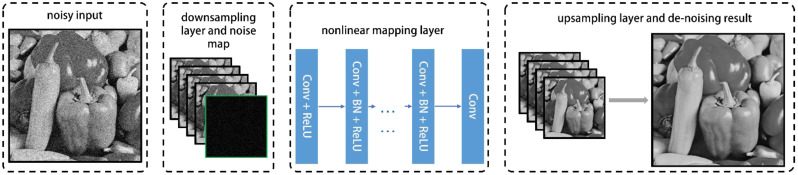
Architecture of FFDNet [22].

Proposed by Zhang et al. in [22], FFDNet is a single discriminative CNN model. Figure 7 shows the architecture of FFDNet. FFDNet consists of “Downsampling layer + Nonlinear mapping layer + Upsampling layer”. The down-sampling layer is a reversible down-sampling operator that reshapes a noisy image into four down-sampled sub-images. At the same time, FFDNet concatenates a tunable noise map with the down-sampled sub-images to form a tensor as the inputs to the non-linear mapping layer. At the non-linear mapping layer, each sub-layer is composed of a specific combination of three types of operations: convolution (Conv) with filter size of 3 × 3, rectified linear units (ReLU), and batch normalization (BN). For the grayscale model, the number of Conv layer is 15 and the number of channels is 64. The noise map varies from 0 to 75 [23]. After the non-linear mapping layer, an upscaling operation is applied in the up-sampling layer as the reverse operator of the down-sampling operator applied in the input stage to produce the estimated clean image with the same shape as the input noisy image.

The training dataset is composed of pairs of input-output patches ${\left\{\left(\left({\tilde{I}}_{j},M_{j}\right),I_{j}\right)\right\}}_{j=0}^{m}$, which are generated by adding AWGN of $\sigma \in [0,75]$ to clean patches $I_{j}$ and build the corresponding noise map $M_{j}$. FFDNet, without a residual learning estimate, can denoise the image directly [23]:

(26) $$F\left(\tilde{I}\right)=\hat{I}$$

thus, the corresponding loss function is [23]:

(27) $$L\left(\theta \right)=\frac{1}{2m}\sum_{j=1}^{m}\|F\left(\left({\tilde{I}}_{j},M_{j}\right);\theta \right)-I_{j}{\|}^{2}$$

where θ is the collection of all learnable parameters. Therefore, the architecture and these additional techniques render this algorithm faster, more efficient, and more versatile than other denoising algorithms.

### 3.3. PnP-ADMM Fixed-Point Convergence for CUP Reconstruction

Ref. [24] demonstrates the fixed-point convergence of the PnP-ADMM algorithm based on the definition of a bounded denoiser and the assumption of bounded gradients.

**Definite** **1.** *Bounded denoiser: A bounded denoiser with a parameter σ is a function $D_{\sigma }:{\mathbb{R}}^{n}\to {\mathbb{R}}^{n}$ such that for any input $x\in {\mathbb{R}}^{n}$ [24]:*

(28) $$\frac{1}{n}\|D_{\sigma }\left(x\right)-x{\|}_{2}^{2}\leq \sigma^{2}C$$

*for some universal constant C, independent of n and σ.*

Bounded denoisers are a weak condition that we expect most denoisers to have. Next, we show that bounded gradients also hold in the CUP reconstruction problem. In the problem of CUP reconstruction, the gradient of $f\left(x\right)$ is:

(29) $$▽f\left(x\right)=H^{⊤}Hx-H^{⊤}y$$

where H is a block-diagonal matrix with element distribution $\left\{0,1\right\}$, all elements of observation y are non-negative, so the result of $H^{⊤}y$ is non-negative. $H^{⊤}Hx$ can be viewed as a weighted sum of x, so $\|H^{⊤}Hx{\|}_{2}\leq n_{x}n_{y}N\|x{\|}_{2}$, since all elements in x are normalized to be between 0 and 1, the inequality can be simplified to: $\|H^{⊤}Hx{\|}_{2}\leq n_{x}n_{y}N$. Therefore, the assumption of constant M, $\|▽f\left(x\right){\|}_{2}\leq M$, bounded gradient holds in the problem of CUP reconstruction. According to the proof of [24], the CUP reconstruction algorithm based on PnP-ADMM has fixed-point convergence, that is, there is $\left(x^{*},v^{*},u^{*}\right)$, and when k→∞, we have:

(30) $$\|x^{\left(k\right)}-x^{*}{\|}_{2}\to 0$$

(31) $$\|v^{\left(k\right)}-v^{*}{\|}_{2}\to 0$$

(32) $$\|u^{\left(k\right)}-u^{*}{\|}_{2}\to 0$$

## 4. Experiment Results

### 4.1. PSNR and SSIM on Simulation Datasets

In order to test the reconstruction ability of PnP-FFDNet, eight frames of data were selected from the benchmark datasets *runner, kobe, traffic, drop,* and *crash* [25] to be compressed and encoded in the way of CUP data compression, and then PnP-ADMM was used for reconstruction. A comparison is made with the CUP reconstruction algorithm TwIST used in [4] and other denoising algorithms used in the algorithmic framework of PnP-ADMM: TVD/BM3D/IRCnn/DnCnn. The computing platform configuration parameters we used are as follows: CPU is 12th Gen Intel(R) Core(TM) i7-12700H 2.70 GHz, GPU is NVIDIA GeForce RTX 3060 laptop GPU.

The benchmark datasets used in the experiment are all 256 × 256 × 8, the size of each frame is 256 × 256, and the total number of frames is eight frames. In all subsequent experiments, the offset $s_{0}$ of each frame is 1. The dimension of the simulated observation data obtained through the data compression model of CUP is 263 × 256. The encoding mask size is 256 × 256, its elements $\left\{0,1\right\}$ are randomly distributed, and the sampling rate is 50%. The regular term of the TwIST algorithm selects the total variation (TV) norm of the image, and the denoising algorithm selects the TVD. Based on the actual test experience, when the parameters manually adjusted in the algorithm are set as follows, a better CUP reconstruction effect can be achieved. For the TwIST algorithm, the regularization parameter is set to 0.05 and the loop is excited when the error of the objective function of two adjacent loops is less than 1e-5. The regularization parameter ρ in the PnP-ADMM algorithm is set to 1, and the iteration is excited when $\Delta_{k+1}$ is less than or equal to 1e-3, where $\Delta_{k+1}$ is [24]:

(33) $$\Delta_{k+1}=\frac{1}{\sqrt{n}}\left(\|x^{\left(k+1\right)}-x^{\left(k\right)}{\|}_{2}+\|v^{\left(k+1\right)}-v^{\left(k\right)}{\|}_{2}+\|u^{\left(k+1\right)}-u^{\left(k\right)}{\|}_{2}\right)$$

**Figure 8:**
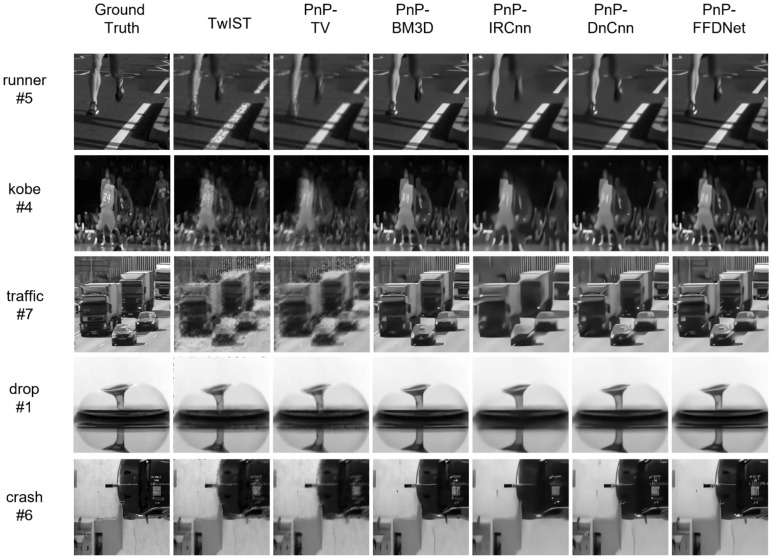
Reconstruction performance of different reconstruction algorithms.

The experiment uses PSNR and SSIM to evaluate the reconstruction performance. PSNR is based on the error between the reconstructed image and the corresponding pixel of the original image. SSIM measures the structural similarity between the reconstructed image and the original image from three aspects: brightness, contrast, and structure. Table 1, Table 2 and Table 3 summarize the PSNR, SSIM, and execution time, respectively of the reconstruction results of the six algorithms. As learning-based denoising algorithms are implemented based on the open-source framework Pytorch, these denoisers can use GPUs to accelerate forward inference. “use GPU” in Table 3 represents the result of the algorithm using GPU-accelerated computing. Figure 8 shows the reconstruction performance of different algorithms on the benchmark dataset.

From the statistical results in Table 1, Table 2 and Table 3, it can be intuitively seen that PnP-FFDNet and PnP-BM3D have the best reconstruction performance. PnP-FFDNet greatly reduces the time required for reconstruction without losing the reconstruction performance. Although other algorithms (such as PnP-TV/PnP-IRCnn/PnP-DnCnn) have advantages in reconstruction time, they sacrifice reconstruction efficiency. Compared with PnP-BM3D, the execution time of PnP-FFDNet is reduced by an average of 93% on the CPU and 96% on the GPU, but the PSNR and SSIM metrics are very similar.

Figure 8 shows the reconstruction performance with different reconstruction algorithms, and selected one frame from eight frames of data for comparison. Among them, the drop data has fewer details than the other four data images, so the six algorithms have good reconstruction performance. For traffic data with more image texture, although the performance of the six reconstruction algorithms is relatively poor, the reconstruction results of the PnP-FFDNet and PnP-BM3D algorithms have clearer contours and less noise.

### 4.2. The Performance of PnP-FFDNet on Data with Different Compression Ratios

In order to test the reconstruction performance of the algorithm with different compression ratios, different frame numbers were intercepted from the drop dataset for CUP data compression encoding, and the PnP-FFDNet algorithm and the PnP-BM3D algorithm were selected for comparison. The data compression ratio R is defined as:

(34) $$R=\frac{size\left(x\right)}{size\left(y\right)}=\frac{n_{x}n_{y}N}{Ln_{y}}=\frac{n_{x}N}{L}=\frac{n_{x}N}{n_{x}+s_{0}\left(N-1\right)}$$

Since the size of each frame in the *drop* dataset is 256 × 256 and $s_{0}$ is 1, the CUP data compression ratio R can be simplified as:

(35) $$R=\frac{256N}{N+255}$$

The larger the number of frames selected for compression coding, the larger the data compression ratio.

**Figure 9:**
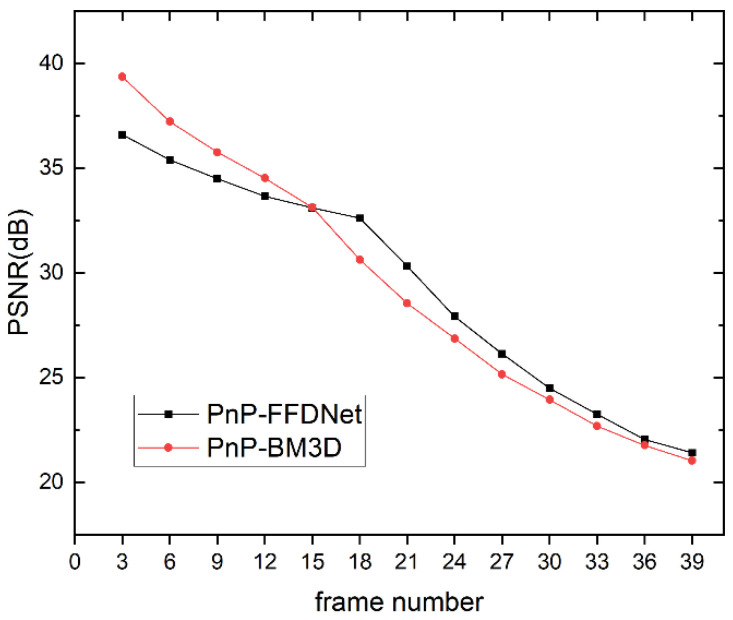
Line chart of PSNR of the reconstruction results of PnP-FFDNet and PnP-BM3D when the number of CUP compressed frames increases.

**Figure 10:**
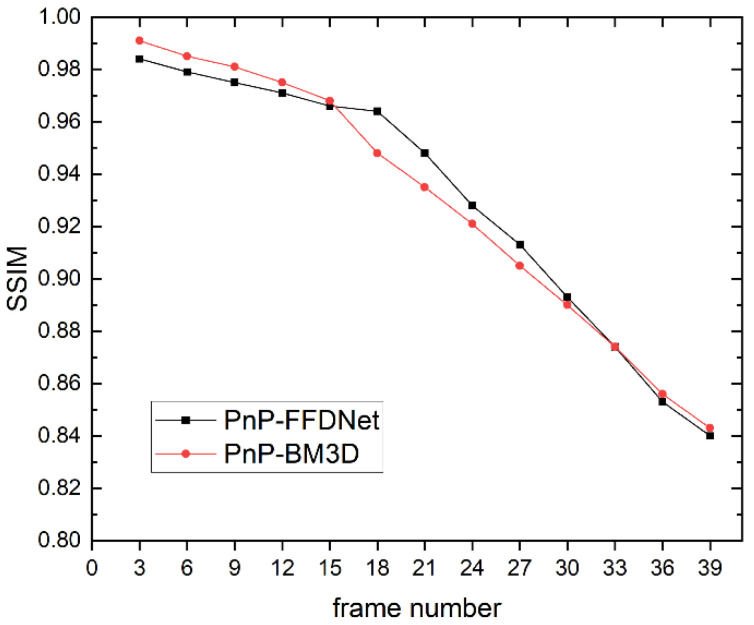
Line chart of SSIM of the reconstruction results of PnP-FFDNet and PnP-BM3D when the number of CUP compressed frames increases.

**Figure 11:**
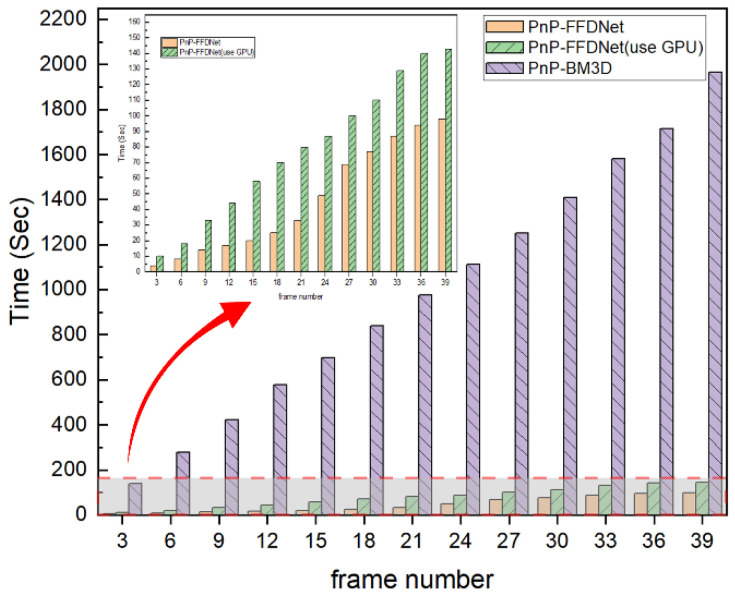
Histogram of execution time of PnP-BM3D, PnP-FFDNet, and PnP-FFDNet (use GPU) when the number of compressed frame increases. The subplot in Figure 7 is an enlarged histogram of the red dashed area.

As shown in Figure 9, Figure 10 and Figure 11, when the data compression rate R increases, our experimental results show that the reconstructed metrics PSNR and SSIM both decrease. When the compression ratio is small, the reconstruction effect of PnP-BM3D is slightly better than that of PnP-FFDNet, but as the compression ratio R increases, the performance of the two algorithms in PSNR and SSIM is close, and even when the compression ratio R is at a certain value, PnP-FFDNet performs better than PnP-BM3D on PSNR and SSIM. However, when the compression ratio R increases, the time consumption of the PnP-BM3D algorithm increases linearly. When the number of compressed frames is 39, the algorithm takes half an hour to complete the reconstruction, while PnP-FFDNet completes the reconstruction in one minute, due to the high efficiency of the algorithm itself and the parallel acceleration of the GPU. The inference speed of FFDNet is greatly accelerated due to the use of down-sampling techniques to reduce the computational load. Therefore, even in CPU, PnP-FFDNet is significantly faster than PnP-BM3D. Furthermore, accelerating the inference phase of the network with the help of GPU parallel computing is also one of the reasons why the PnP-FFDNet algorithm executes faster.

### 4.3. Performance of PnP-FFDNet on Real Data

In order to test the performance of the PnP-FFDNet algorithm on real CUP experimental data, key frames were extracted from the video file of the laser pulse reflection process in the Supplementary Material of [4], and the RGB image was converted into a grayscale image to form a 320 × 320 × 16 original image data, and 335 × 320 observation data were obtained through the CUP data compression model. Using TwIST and PnP-TV/BM3D/IRCnn/DnCnn/FFDNet, we performed algorithm reconstruction experiments.

**Figure 12:**
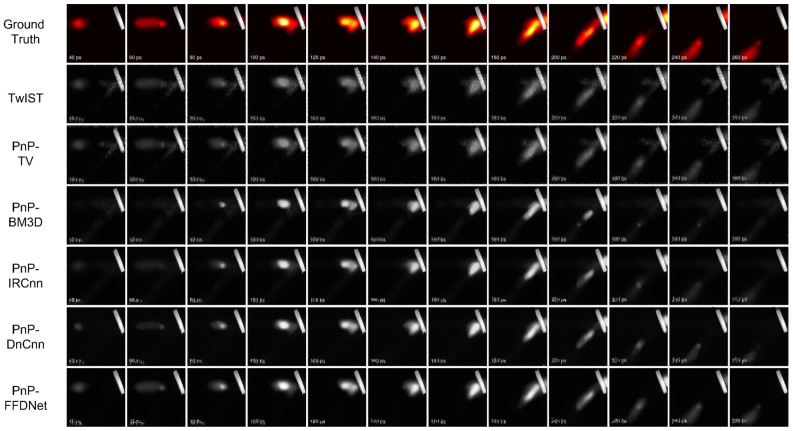
Reconstruction performance of different reconstruction algorithms on real data.

Figure 12 shows the reconstruction results with six algorithms on the real experimental data of CUP. The algorithm reconstructs a total of 16 frames, and selects 12 consecutive frames with clear pulsed laser graphics for comparison. Due to the process of compression sampling, only part of the information is sampled, the spatial resolution of the image reconstructed by the algorithm is low, and so the text in the image has more details and, therefore, cannot be reconstructed well. From the image comparison of the reconstruction results, it can be seen that the laser reflection process of the PnP-FFDNet reconstruction results is the closest to the ground truth, and the reconstructed visual effects, evaluation indicators (PSNR, SSIM), and operating efficiency all exceed the other algorithms.

Table 4 shows the reconstruction indicators. Compared with TwIST, PnP-TV and PnP-BM3D, PSNR is improved by 3.95 dB, 4.61 dB, and 1.85 dB, respectively, and SSIM is improved by 0.07, 0.1, and 0.02, respectively. Under the premise of obtaining a better reconstruction effect, PnP-IRCnn/PnP-DnCnn/PnP-FFDNet require relatively less reconstruction time. The reconstruction effects of the algorithms using the learning-based denoiser are similar on the metric PSNR, and PnP-FFDNet is the best for the metric SSIM. Although the running time of the PnP-TV algorithm is the shortest, its reconstruction effect is similar to that of TwIST. The performance on real datasets shows that PnP-FFDNet has excellent performance in reconstruction effect and running efficiency.

## 5. Conclusions

This paper proposes a CUP reconstruction algorithm based on the combination of the PnP-ADMM framework and the convolutional neural network denoising algorithm FFDNet. The reconstruction methods were performed on the benchmark dataset and the real data of the CUP experiment. The results show that the proposed algorithm performs better on the PSNR and SSIM. Since the inference of the convolutional neural network can be performed in parallel using the GPU, the execution time of PnP-FFDNet is greatly reduced compared to PnP-BM3D, without losing the re-estimation effect. In the popular CUP experimental configuration, the data depth is often between 150–1500. The proposed method can greatly speed up the reconstruction process. and is a practical and efficient CUP reconstruction algorithm.

## Figures and Tables

**Table 1:** The average PSNR (dB) results.

Algorithm	Runner	Kobe	Traffic	Drop	Crash	Average
TwIST	24.52	25.42	19.16	29.39	24.54	24.70
PnP-TV	23.29	23.89	19.55	29.72	24.57	24.20
PnP-BM3D	30.59	29.15	23.77	36.40	26.01	29.18
PnP-IRCnn	25.86	24.83	21.13	29.90	24.56	25.24
PnP-DnCnn	28.12	27.54	22.27	32.60	24.94	27.09
PnP-FFDNet	29.68	28.86	23.19	34.89	25.21	28.37

**Table 2:** The average SSIM results.

Algorithm	Runner	Kobe	Traffic	Drop	Crash	Average
TwIST	0.82	0.82	0.58	0.92	0.82	0.79
PnP-TV	0.82	0.84	0.68	0.95	0.87	0.83
PnP-BM3D	0.95	0.92	0.84	0.98	0.90	0.92
PnP-IRCnn	0.87	0.82	0.73	0.94	0.85	0.84
PnP-DnCnn	0.91	0.85	0.80	0.96	0.87	0.88
PnP-FFDNet	0.93	0.92	0.82	0.98	0.88	0.91

**Table 3:** The execution time (second).

Algorithm	Runner	Kobe	Traffic	Drop	Crash	Average
TwIST	41	67	47	177	104	87
PnP-TV	10	7	9	6	8	8
PnP-BM3D	378	402	387	436	379	396
PnP-IRCnn	33	35	36	34	35	35
PnP-IRCnn (use GPU)	10	11	12	10	11	11
PnP-DnCnn	75	79	80	78	82	79
PnP-DnCnn (use GPU)	14	14	15	14	14	14
PnP-FFDNet	27	27	27	26	27	27
PnP-FFDNet (use GPU)	12	13	12	12	10	12

**Table 4:** Results of PSNR (dB), SSIM, execution time (s) and execution time (s) (use GPU) for different algorithms on real data.

Algorithm	PSNR	SSIM	Execution Time (s)	Execution Time (s) (Use GPU)
TwIST	21.17	0.88	270	-
PnP-TV	21.04	0.85	33	-
PnP-BM3D	23.80	0.93	1124	-
PnP-IRCnn	25.67	0.91	103	26
PnP-DnCnn	25.27	0.87	230	40
PnP-FFDNet	25.65	0.95	85	43

## Data Availability

Not applicable.
